# Acute phase proteins levels in horses, after a single carbohydrate overload, associated with cecal alkalinization

**DOI:** 10.3389/fvets.2023.1043656

**Published:** 2023-02-02

**Authors:** Isabela Peixoto Rabelo, Vanessa Barroco de Paula, Caio Carvalho Bustamante, André Marcos Santana, Daniela Gomes da Silva, Amanda Cristina Baldassi, Paulo Aléscio Canola, Carlos Augusto Araújo Valadão

**Affiliations:** ^1^Department of Veterinary Internal Medicine and Surgery, School of Agricultural and Veterinarian Sciences, São Paulo State University (Unesp), Jaboticabal, São Paulo, Brazil; ^2^Department of Veterinary Medicine, Maringá State University, Umuarama, Paraná, Brazil; ^3^Department of Agricultural, Livestock and Environmental Biotechnology, School of Agricultural and Veterinarian Sciences, São Paulo State University (Unesp), Jaboticabal, São Paulo, Brazil

**Keywords:** electrophoresis, gastrointestinal buffering, protein fractionation, SIRS, acute phase response

## Abstract

**Introduction:**

Horses submitted to carbohydrate overload can develop laminitis due to changes in cecal pH and microbiota, followed by an increase in transmural absorption of luminal content, including bacterial toxins. In response to acute injury there is hepatic overproduction of several proteins known as acute phase proteins (APP). Few studies have evaluated protein fractionation to characterize the inflammatory response in acute laminitis. The aim of this study was to test the viability of an experimental model to induce acute laminitis, using a single carbohydrate overload, and the influence of a buffering solution on the development of the disease; also, study the kinetics of APP during acute laminitis, as well as the correlation between these proteins and clinical signs associated to this syndrome.

**Methods:**

Ten healthy horses were divided in a factorial and randomized way into four groups (*n* = 5): control group (CG), starch group (SG), buffer group (BG), and starch C buffer group (SBG). They were evaluated at seven times (T0h, T4h, T8h, T12h, T24h, T48h, and T72h), which included clinical evaluation and blood sample collection. Total serum protein and albumin concentrations were determined by colorimetry and the other APP by polyacrylamide gel electrophoresis containing sodium dodecyl sulfate and commercial ELISA kits. Data were analyzed by two-way ANOVA, followed by Tukey's test (*p* < 0.05). The correlation between clinical signs and APP were verified using the Pearson's correlation coefficient.

**Results and discussion:**

40% of the animals from SG and 60% from SBG developed clinical laminitis. A single administration of buffer solution was not able to prevent clinical signs of laminitis. There was no difference between groups on total serum protein, albumin, serum amyloid A and C-reactive protein concentrations (*p* > 0.05). Transferrin, considered a negative APP, showed a positive response pattern in SG and SBG. Ceruloplasmin had a positive correlation with Obel grade, heart rate on animals from SGB and number of steps on horses submitted to starch overload (SG and SBG). Ceruloplasmin, α-1-antitrypsin and haptoglobin concentrations increased in SBG, suggesting an inflammatory response in animals of this group. Changes in clinical parameters were also more evident in the SBG, corroborating the protein fractionation findings.

## 1. Introduction

Equine laminitis is a devastating condition characterized, in most cases, by excruciating pain and irreversible changes in the digit, incapacitating animals for their intended use but also resulting in death, with economic or affective losses. Despite all the efforts, treatment is often inadequate, many horses that recover do not return to their original athletic activity (1). For these reasons, establishment of early or preventive treatments is imperative to reduce disease occurrence or severity (2).

Laminitis development due to alimentary factors has been well documented (3). Excessive intake of carbohydrates (CHO) increases cecal fermentation, exceeding buffering capacity, which results in cecal pH acidification, changes in microbiota, accumulation of bacterial byproducts (e.g., endotoxins, exotoxins) that are absorbed, triggering a systemic inflammatory response (4). The single CHO overload model (oligofructose) is a reliable method to experimentally induce laminitis in healthy horses. Furthermore, it has been shown that administration of a buffering solution intracecally can minimize changes in the microbiota, preventing the development of SIRS and associated laminitis (5).

Identification of an inflammatory response preceding initial signs of laminitis is fundamental, since the appearance of these signs generally reflect irreversible changes in the lamellar tissue (2, 6). It has been proposed that protein fractionation can be useful to detect the onset of the inflammatory response, before the appearance of other systemic signs (7). Signals from stressors factors are detected by the hypothalamus, activating the hypothalamic-pituitary-adrenal and sympathoadrenal axes, which lead to the release of glucocorticoids and catecholamines, respectively, that, through the induction of pro-inflammatory cytokines by macrophages and lymphocytes, promote the production of acute phase proteins in hepatocytes and in the circulation in stressed animals. Therefore, serum proteins are involved in the regulation of inflammation and protection against infections, and therefore, serum protein profiling has been used to monitor the severity of inflammatory responses in veterinary medicine (8–12).

Acute phase proteins (APP) such as serum amyloid A and fibrinogen are often routinely used in equine practice to assess infection, severity of infection, but also progression and recovery (13–16). These proteins represent a portion of many other proteins produced by the liver (e.g., ceruloplasmin, haptoglobin, C reactive protein, and α-1-antitrypsin) in response to insults. The rate of production of some APP has been associated with laminitis in horses (7). Considering the recent discoveries about the role of APP in inflammation and laminitis (17), further studies about the establishment of this syndrome and early APP involvement needs to be conducted.

Our main goals were: (1) verify the effectiveness of single carbohydrate overload in promoting laminitis; (2) assess the effect of a buffer solution administered intracecally on the development of laminitis; and (3) study the kinetics of acute phase proteins during acute laminitis, as well as the correlation between these proteins and clinical signs associated to acute laminitis.

## 2. Materials and methods

### 2.1. Animals and study design

Ten healthy horses, geldings and females, aged 6 ± 3 years, weighting 350 ± 50 kg, were included in the study. Horses were considered healthy based on physical evaluation, hemogram and biochemistry profile, and were under a regular deworming and vaccination program. Horses had no history of disease for at least 6 months prior to the study, were not lame and had no history or clinical evidence of laminitis. Horses had a body condition score of 6, with no indication of insulin dysregulation, although baseline insulin concentrations were not measured. All horses were submitted to typhlopexy 30 days before the experiment (18). They were housed in the Equinoculture Sector at FCAV/Unesp-Jaboticabal Campus and received daily 2 kg of commercial feed (Selvagem^®^–Agromix—Jaboticabal, SP, Brazil), 4 kg of coast-cross hay (*Cynodon dactylon*) and water *ad libitum*. During the 3 days of experimental period (T0h to T72h), horses were kept in individual stalls, without access to the paddock, and fed the same way as described.

The starch overload consisted in a 17.6 g/kg BW dose of powdered corn starch diluted in 1 kg per liter of water (19). Buffer solution containing 3.5 g of aluminum hydroxide, 65.6 g of magnesium hydroxide and 1.2 g of simethicone, was diluted in half amount of water calculated for the starch overload. Horses were randomly allocated into 4 groups (*n* = 5) in a factorial 2 × 2 way:

- **Control group (CG)**: water administration *via* nasogastric tube (NGT) and, after 8 h, intracecal (IC) saline solution.
- **Starch group (SG)**: starch administration *via* NGT and, after 8 h, IC saline solution.
- **Buffer group (BG)**: water administration *via* NGT and, after 8 h, IC buffer solution.
- **Starch/Buffer group (SBG)**: starch administration *via* NGT and, after 8 h, IC buffer solution.

After a 15 days' interval, horses from CG and BG groups received SBG and SG treatments, respectively.

Clinical and behavior parameters were evaluated at seven time points: Immediately before the administration of starch/water *via* NGT (T0h), and 4 (T4h), 8 (T8h), 12 (T12h), 24 (T24h), 48 (T48h), and 72 h (T72h) after the administration of starch/water *via* NGT. Clinical parameters included heart rate (bpm), rectal temperature (°C), dehydration (%), oral mucosal aspect (normal or abnormal), plus digital pulse and hoof temperature (normal or elevated). Behavior was assessed by signs of discomfort, movement, food and water intake and feces production. The gait quality and number of steps on a flat cement floor in a six-meter space were visually evaluated and counted. Signs of locomotion impairment and limb pain were categorized according to Obel (20).

Blood samples were collected *via* jugular venipuncture (BD Vacutainer^®^, Franklin Lakes, NJ, USA) at the same seven time points. The samples were centrifuged for 10 min at 1,000 × g in a temperature-controlled room (21°C) and stored in a −24°C freezer until analyses.

### 2.2. Serum acute phase proteins and immunoglobulin analysis

Total serum protein and albumin concentrations were determined by spectrophotometry in a semi-automatic biochemical analyzer (Labquest—Labtest Diagnóstica SA, Lagoa Santa, MG, Brazil) by biuret (Total Proteins, Ref. 99-250, Labtest Diagnóstica SA, Lagoa Santa, MG, Brazil / bovine albumin standard 4.0 g/dL) and green bromocresol (Albumin, Ref. 19-1/250, Labtest Diagnóstica SA, Lagoa Santa, MG, Brazil/bovine albumin standard 3.8 g/dL) colorimetric methods, respectively. Identification and quantification of APP were obtained through sodium dodecyl sulfate-polyacrylamide gel electrophoresis (SDS-PAGE) (21). Quantitative analysis of the SDS-PAGE was performed using a computerized densitometer (model CS-9301PC, Shimadzu Corporation, Tokyo, Japan). Serum concentrations of C-reactive protein (MyBioSource ELISA Horse C-reactive protein, catalog number MBS020917/intra and inter-assay CV is < 15%) and serum amyloid A (MyBioSource ELISA Horse Serum Amyloid A, catalog number MBS281865/intra-assay CV < 8%/inter-assay < 12%) were performed by ELISA.

Five different band fractions of interest were cut from multiple gels generated by the SDS-PAGE technique. The samples were than submitted to trypsin digestion (22) and filtered in a cellulose acetate membrane according to manufacturer's instruction (Spin-X, Corning). Posteriorly, the samples were analyzed by liquid chromatography coupled to mass spectrometry (LC-MS) using a C18 nano-column (15 cm 2 μm, 100 Å) and a chromatographic gradient of 35 min. Mass spectrometer (Q-Exactive, Thermo Scientific) was operated in the data-dependent acquisition mode in a top 20. Protein identification was carried out by spectral correlation in the Patternlab platform (23) and assigned using NCBI predicted protein database from the species *Equus caballus*. False-discovery rate was adjusted for 1%. This whole analysis was carried out at the Technology Department of the School of Agricultural and Veterinarian Sciences—FCAV/ Unesp-Jaboticabal Campus.

### 2.3. Statistical analysis

Normality was assessed by the Shapiro–Wilk test and data were normally distributed. Therefore, data were subjected to analysis of variance (ANOVA) and Tukey's test for comparisons. Associations between variables was performed using Pearson's correlation. A *p* < 0.05 was considered statistically significant. Statistical software included Statistical Analysis System (SAS) program (SAS, 9.1.3 version, SAS Institute, Cary, NC, USA) and GraphPad Prism (Version 7.0).

## 3. Results

### 3.1. Experimental model for laminitis induction and effect of treatment using buffer solution

All animals in SG had tachycardia after T4h, and by T24h, 40 (2/5) and 60% (3/5) had dehydration and diarrhea, respectively (Table 1). However, alterations in clinical parameters were more expressive in SBG, especially between T24h and T48h. All horses in SBG had evidence of dehydration at T24h and hyporexia at T48h. Throughout the experimental period, horses from CG and BG maintained their heart rate (HR) and body temperature (°C) within the normal range (24), and presented no alterations of gait, hoof temperature, digital pulse, and food and water intake.

**Table 1:** Physical examination findings in horses subjected to carbohydrate overload, untreated and treated with intracecal buffer solution (Starch group—SG and Starch/Buffer group—SBG, respectively), in the seven evaluated moments (T0h, T4h, T8h, T12h, T24h, T48h, and T72h).

**Parameter**	**Unit**	**T0h**	**T4h**	**T8h**	**T12h**	**T24h**	**T48h**	**T72h**
**SG (*****n*** = **5)**								
Average HR	Bpm	39	54	58	54	50	49	59
Average rectal T°	°C	37.2	37.6	37.7	37.7	37.4	37.7	37.5
Dehydration	Animals (%)	0	0	0	20	40	0	0
Hyporexia	Animals (%)	0	0	0	0	0	20	0
Diarrhea	Animals (%)	0	0	0	0	60	40	0
Digital pulse	Animals (%)	0	20	20	20	40	20	0
↑ hoof T°	Animals (%)	0	20	20	0	20	40	20
Lameness	Animals (%)	0	0	0	0	0	40	20
**SBG (*****n*** = **5)**								
Average HR	Bpm	40	49	**4**4	58	54	70	59
Average rectal T°	°C	37.3	37.4	38.2	39.3	38.4	38.1	37.7
Dehydration	Animals (%)	0	0	20	60	100	60	40
Hyporexia	Animals (%)	0	0	40	40	80	100	40
Diarrhea	Animals (%)	0	0	0	60	80	60	0
Digital pulse	Animals (%)	0	0	0	0	80	20	80
↑ hoof T°	Animals (%)	0	0	0	0	60	80	60
Lameness	Animals (%)	0	0	0	0	20	60	60

Results are presented as means.

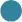
 Altered parameters (24).

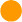
 Signs of laminitis development.

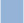
 T0h: carbohydrate overload.

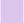
 T8h: buffer solution administration.

Three horses from SBG and two from SG developed laminitis (6, 20, 25), with increased digital pulse and hoof temperature, and lameness (Table 1). 20% (1/5) of animals from SG showed signs of laminitis before the administration of the buffer solution. In SBG, clinical signs were delayed (8 h after starch overload, matching the exact time of the treatment), when compared to SG.

### 3.2. APP kinetics and correlation with clinical parameters during the inflammatory process linked to laminitis syndrome

**Figure 1:**
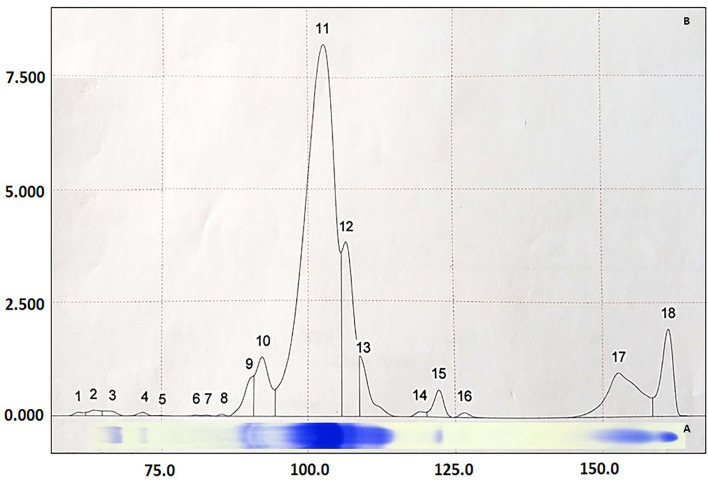
Graphic representation of proteins identified in equine serum by the SDS-PAGE technique **(A)** and quantified by densitometry **(B)**. 18 proteins were found and 8 identified by their molecular weight (kDa): (3) IgA (175 kDa), (4) ceruloplasmin (130 kDa), (9 and 10) transferrin (85 kDa), (11) albumin (65 kDa), (12) α-1-antitrypsin (60 kDa), (13) IgG-HC (55 kDa), (15) haptoglobin (45 kDa) and (17) IgG-LC (29 kDa).

Through SDS-PAGE (Figure 1) and posterior LC-MS confirmation (Table 2), it was possible to identify four APP (followed by its molecular weight in kDa): transferrin (85 kDa), albumin (65 kDa), α-1-antitrypsin (60 kDa), and haptoglobin (45 kDa). Based on ceruloplasmin (Cp) molecular weight, this protein should have been identified in the 130 kDa protein fraction on the SDS-PAGE gel. This section was cut from polyacrylamide gel, submitted to LC-MS (26–28), but it did not bring a reliable identification. Assuming that the location of Cp in the protein fraction matches the literature (26–28), we chose to maintain the results of the alleged ceruloplasmin (aCp) in this study, which was obtained by densitometry analysis from the SDS-PAGE.

**Table 2:** Identification of *Equus caballus* serum proteins by liquid chromatography coupled to mass spectrometry (LC-MS/MS), based on the chemical digestion of five bands of interest in polyacrylamide gels (SDS-PAGE).

**Band no (Figure 1)**	**Identified proteins**	**Identification (access no)**	**Amino acids (no)**	**Single peptides found (no)**	**Coverage (%)**	**Abundance (%)**
4	Alpha-2-macroglobulin	XP_001499173.3	1,476	12	9.2	2.2
	Immunoglobulin lambda-1 (LC)	XP_023503887.1	234	3	13.7	1.6
9 e 10	Serotransferrin precursor	NP_001075415.2	706	34	51.1	57.1
	Complement C4-A	XP_001492943.1	1,744	13	8.3	1.0
	Antithrombin-III	XP_014594947.1	463	5	10.2	1.0
	Immunoglobulin lambda-1 (LC)	XP_023503887.1	234	3	13.7	1.8
	Inhibitor of carbonic anhydrase	XP_023476722.1	700	3	6.9	2.0
11	Serum albumin precursor	NP_001075972.1	607	52	70.8	90.1
	Liver carboxylesterase	XP_014593950.1	550	14	33.8	3.0
	Alpha-1-antiproteinase 2-like precursor	NP_001304178.1	421	7	20.0	2.9
12	Serum albumin precursor	NP_001075972.1	607	10	14.5	43.9
	Alpha-2-macroglobulin	XP_001499173.3	1,476	8	6.0	2.1
	Alpha-1-antiproteinase 2-like precursor	NP_001304178.1	421	5	14.3	32.1
	Antithrombin-III	XP_014594947.1	463	5	11.0	15.2
15	Haptoglobin	XP_001497860.1	347	4	11.5	91.0
	Serum albumin precursor	NP_001075972.1	607	2	3.0	6.3

The shade color was used to highlight, among all proteins identified in the band of interest, the one that matched the result obtained by the SDS_PAGE technique.

**Figure 2:**
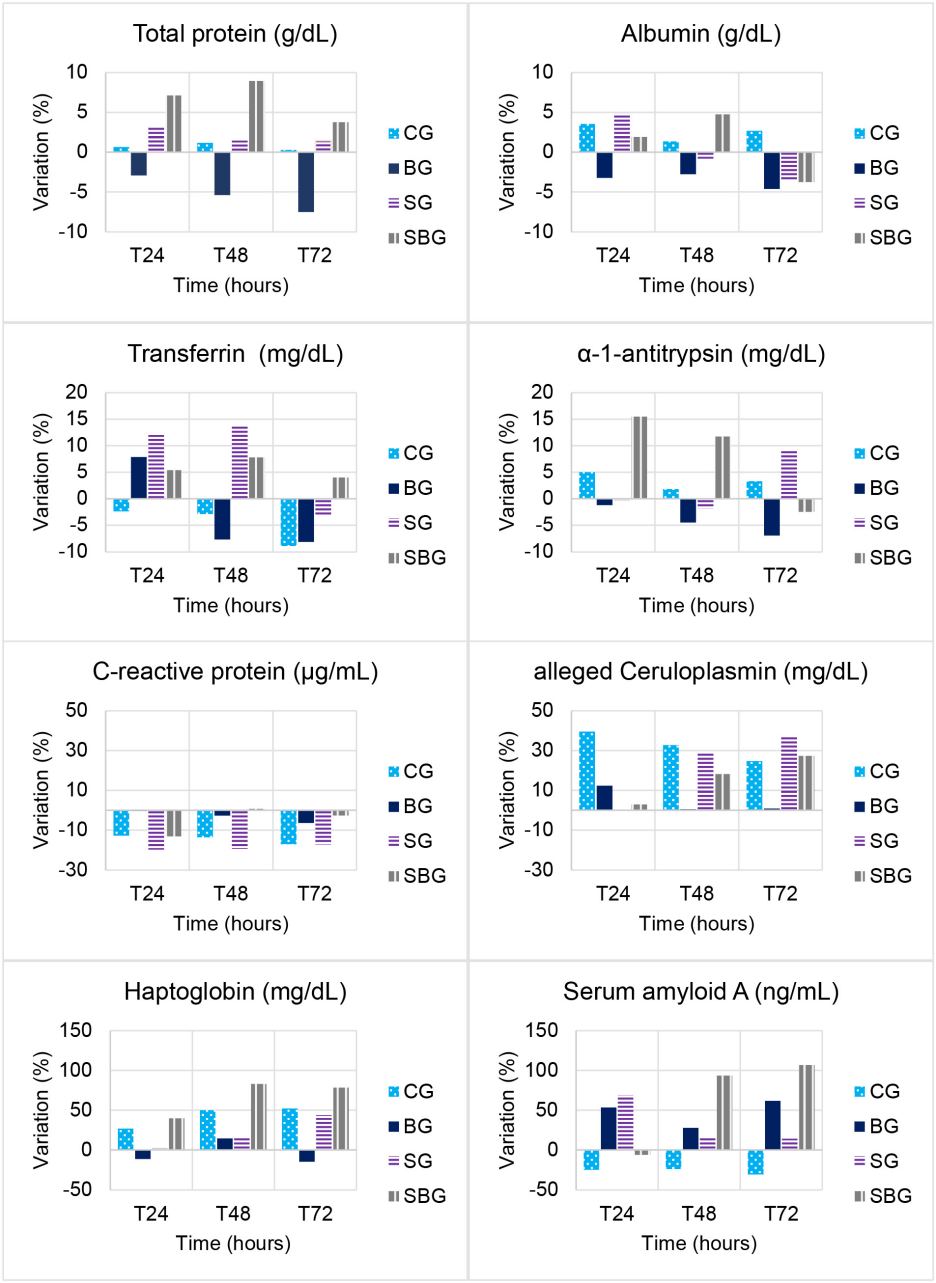
Variation (%) from baseline over time (T0h, T4h, T8h, T12h, T24h, T48h, and T72h) in total serum protein, albumin, transferrin, α-1-antitrypsin, C-reactive protein, alleged ceruloplasmin, haptoglobin and serum amyloid A concentrations in horses submitted to experimental laminitis (CG, control; BG, buffer; SG, starch; SBG, starch/buffer).

As detailed in Table 3, the only APP that varied through time, when considering each group separately, was haptoglobin (Hp), especially after T48h in SG and SBG (*p* < 0.05). Between SG and CG, there was no difference (*p* > 0.05) in the levels of aCp and α-1-antitrypsin (α-1-AT). In contrast, SBG had increased serum concentrations of these three APP, when compared with CG, at least in one of the last three moments (*p* < 0.05), reinforcing the findings of clinical examination. Only Hp varied between animals of BG and SBG at T24h, T48h, and T72h (*p* < 0.05). This APP also differed between the BG and SG horses at T72h (*p* < 0.05). Serum transferrin (Trf) concentration was higher in SG, when compared to CG, at T8h, T24h, and T48h (Table 3). Among the SG and SBG, transferrin elevation was higher at T48h in the group that did not receive intracecal buffer solution (Figure 2). There was no difference in serum concentrations of total protein, albumin (Alb), C-reactive protein (CRP) and serum amyloid A (SAA) among time and groups (*p* > 0.05).

**Table 3:** Mean ± standard deviation of serum concentrations of total protein, albumin, α-1-antitrypsin, C-reactive protein, transferrin, ceruloplasmin, haptoglobin and serum amyloid A of horses in each experimental group (CG, control; BG, buffer; SG, starch; SBG, starch/buffer); during the seven evaluated moments (T0h, T4h, T8h, T12h, T24h, T48h, and T72h).

**Total protein (g/dL)^1^**							
	**T0h**	**T4h**	**T8h**	**T12h**	**T24h**	**T48h**	**T72h**
CG	7.89 ± 0.40	7.59 ± 0.40	7.64 ± 0.36	7.57 ± 0.32	7.94 ± 0.38	7.98 ± 0.31	7.91 ± 0.43
BG	8.51 ± 0.15	8.21 ± 0.57	7.86 ± 0.84	8.59 ± 0.63	8.26 ± 0.26	8.05 ± 0.40	7.87 ± 1.24
SG	8.38 ± 0.57	8.00 ± 0.40	8.16 ± 1.08	7.73 ± 0.67	8.65 ± 0.35	8.50 ± 0.78	8.50 ± 0.61
SBG	8.30 ± 0.63	NE	NE	NE	8.89 ± 0.80	9.04 ± 0.66	8.61 ± 0.81
**Albumin (g/dL)** ^1^							
CG	2.27 ± 0.26	2.29 ± 0.26	2.23 ± 0.24	2.20 ± 0.25	2.35 ± 0.32	2.30 ± 0.30	2.33 ± 0.33
BG	2.16 ± 0.15	2.09 ± 0.13	2.13 ± 0.15	2.00 ± 0.18	2.09 ± 0.35	2.10 ± 0.21	2.06 ± 0.19
SG	2.29 ± 0.07	2.23 ± 0.21	2.35 ± 0.26	2.22 ± 0.20	2.40 ± 0.24	2.27 ± 0.30	2.21 ± 0.14
SBG	2.12 ± 0.16	NE	NE	NE	2.16 ± 0.19	2.22 ± 0.24	2.04 ± 0.36
**Transferrin (mg/dL)** ^2^ **–MW: 85 kDa**							
CG	436 ± 125	420 ± 114	426 ± 111^A^	430 ± 105^A^	426 ± 86.8^A^	424 ± 68.6^A^	397 ± 87.5
BG	544 ± 81.6	523 ± 63.4	512 ± 66.3^AB^	580 ± 83.0^B^	587 ± 146^B^	502 ± 114^AB^	499 ± 104
SG	523 ± 65.0	504 ± 54.0	571 ± 154^B^	526 ± 117^AB^	585 ± 163^B^	595 ± 145^B^	505 ± 74.5
SBG	413 ± 79.9	NE	NE	NE	435 ± 104^A^	445 ± 51.8^A^	430 ± 49.8
**Alleged ceruloplasmin (mg/dL)** ^3^ **–MW: 130 kDa**							
CG	17.8 ± 10.0^A^	22.6 ± 2.42^A^	23.5 ± 4.56	22.8 ± 1.62^A^	24.8 ± 3.65^A^	23.6 ± 2.24^A^	22.2 ± 6.34^A^
BG	32.8 ± 4.42^B^	38.5 ± 3.66^B^	34.6 ± 7.70	36.6 ± 4.83^B^	36.8 ± 4.58^AB^	33.0 ± 8.67^AB^	33.1 ± 6.55^A^
SG	25.4 ± 5.78^AB^	27.0 ± 3.49^AB^	24.5 ± 7.10	24.7 ± 6.44^AB^	25.4 ± 7.44^A^	32.6 ± 10.1^AB^	34.7 ± 6.86^A^
SBG	37.9 ± 5.63^B^	NE	NE	NE	38.9 ± 5.98^B^	44.7 ± 10.0^B^	48.2 ± 15.1^B^
α**-1-antitrypsin (mg/dL)**^2^**–MW: 60 kDa**							
CG	685 ± 139	725 ± 88.5	736 ± 90.8	749 ± 111	718 ± 164^A^	697 ± 120^A^	707 ± 162
BG	814 ± 94.1	856 ± 15.8	671 ± 135	783 ± 21.7	804 ± 21.3^AB^	777 ± 18.5^AB^	757 ± 3.75
SG	798 ± 190	681 ± 278	761 ± 285	700 ± 95.1	795 ± 172^AB^	783 ± 270^AB^	871 ± 341
SBG	904 ± 301	NE	NE	NE	1,044 ± 419^B^	1,009 ± 451^B^	881 ± 421
**Haptoglobin (mg/dL)** ^2^ **–MW: 45 kDa**							
CG	31.2 ± 17.4	34.6 ± 19.7^A^	34.8 ± 20.9^A^	33.3 ± 18.7^A^	39.5 ± 23.2^A^	46.8 ± 21.4^A^	47.4 ± 19.9^A^
BG	64.8 ± 30.0	57.2 ± 21.9^AB^	55.8 ± 20.5^AB^	57.8 ± 15.4^AB^	57.5 ± 21.4^AB^	74.2 ± 31.0^B^	55.3 ± 21.7^A^
SG	71.8 ± 33.7^a^	68.8 ± 20.7^Ba^	71.3 ± 30.9^Ba^	65.1 ± 12.4^Ba^	73.2 ± 19.2^BCa^	83.1 ± 17.9^Bab^	103 ± 16.7^Bb^
SBG	66.4 ± 13.6^a^	NE	NE	NE	92.7 ± 30.0^Cab^	121 ± 29.4^Cc^	118 ± 13.2^Bbc^
**Serum amyloid A (ng/mL)** ^4^							
CG	332 ± 13.0	294 ± 71.0	328 ± 101	200 ± 73.6	248 ± 86.9	251 ± 146	229 ± 63.3
BG	219 ± 152	316 ± 175	316 ± 100	357 ± 387	336 ± 253	280 ± 246	354 ± 175
SG	267 ± 414	199 ± 239	301 ± 343	733 ± 793	450 ± 572	308 ± 417	304 ± 459
SBG	44.0 ± 31.7	NE	NE	NE	41.2 ± 13.6	85.0 ± 80.3	90.8 ± 51.7
**C reactive protein (**μ**g/mL)**^4^							
CG	1.88 ± 0.23	1.95 ± 0.46	1.60 ± 0.24	1.65 ± 0.23	1.64 ± 0.30	1.62 ± 0.49	1.56 ± 0.12
BG	1.80 ± 0.53	1.62 ± 0.13	1.60 ± 0.31	1.75 ± 0.31	1.79 ± 0.26	1.75 ± 0.42	1.68 ± 0.42
SG	1.91 ± 0.70	1.78 ± 0.97	3.08 ± 3.18	1.53 ± 0.63	1.53 ± 0.54	1.53 ± 0.56	1.57 ± 0.63
SBG	2.23 ± 1.85	NE	NE	NE	1.94 ± 1.11	2.25 ± 1.46	2.17 ± 1.48

NE, not evaluated; MW, molecular weight.

Values obtained by (1) spectrophotometry and absorbance reading; (2) SDS-PAGE, densitometer reading, identification by MW and confirmation by LC-MS; (3) SDS-PAGE, densitometer reading and identification by MW; (4) ELISA and absorbance reading.

Different lower-case letters indicate differences within the same group and different upper case letters indicate differences between groups by Tukey's test (p < 0.05).

As represented in Table 4, aCp showed a positive correlation with number of steps in SG and SBG. In SBG, this protein also correlated positively with Obel grade and HR. In SG, number of steps had a negative correlation with α-1-AT and a positive one with Alb.

**Table 4:** Pearson's correlation coefficient (*r*) and significance (*p*), from serum concentrations of total protein and acute phase proteins, with the physiological parameters evaluated in horses subjected to carbohydrate overload, untreated and treated with intracecal buffer solution (SG, starch group; SBG, starch/buffer group, respectively).

**Physiological**	**TP**		**Alb**		**aCp**		**Trf**		α**-1-AT**		**Hp**		**SAA**		**CRP**	
**Parameters**	**r**	**p**	**r**	**p**	**r**	**p**	**r**	**p**	**r**	**p**	**r**	**p**	**r**	**p**	**r**	**p**
**SG (*****n*** = **5) proteins**																
No of steps	0.1	0.527	0.4	**0.014**	0.5	**0.005**	0.1	0.453	−0.5	**0.005**	−0.1	0.668	−0.2	0.330	−0.2	0.174
HR	0.0	0.890	−0.2	0.212	0.0	0.798	0.1	0.422	0.3	0.085	0.3	0.065	0.2	0.355	0.0	0.815
T°C	0.1	0.454	0.0	0.929	0.1	0.608	−0.1	0.419	0.2	0.364	−0.1	0.632	0.1	0.503	0.1	0.675
Dehydration	0.0	0.973	0.0	0.913	−0.2	0.365	0.0	0.818	0.1	0.480	−0.1	0.643	−0.2	0.354	−0.1	0.516
Obel grade	0.0		0.0		0.0		0.0		0.0		0.0		0.0		0.0	
**SBG (*****n*** = **5) proteins**																
Obel grade	−0.2	0.327	0.1	0.622	0.5	**0.033**	0.1	0.642	−0.6	0.096	0.2	0.461	0.1	0.747	0.3	0.219
HR	0.0	0.968	0.2	0.439	0.6	**0.009**	0.2	0.305	−0.4	0.283	0.2	0.316	0.2	0.379	0.0	0.967
No of steps	0.1	0.657	0.0	0.934	0.6	**0.012**	0.1	0.680	−0.3	0.403	0.4	0.104	0.3	0.156	0.2	0.446
T°C	0.4	0.058	0.1	0.543	0.1	0.629	0.2	0.510	0.2	0.659	0.3	0.269	0.1	0.803	0.0	0.868
Dehydration	0.1	0.830	0.4	0.080	0.3	0.278	0.4	0.127	−0.5	0.213	0.0	0.985	0.0	0.939	0.2	0.406

HR, heart rate; T°C, rectal temperature; TP, total protein; Alb, albumin; aCp, alleged ceruloplasmin; Trf, transferrin; α-1-AT, α-1-antitrypsin; Hp, haptoglobin; SAA, serum amyloid A; CRP, C reactive protein. Correlation (29): 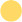 Negative (r < 0); 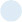 Weak (0.1 ≤ r ≤ 0.3); 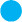 Moderate (0.4 ≤ r ≤ 0.6); 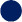 Strong (0.7 ≤ r ≤ 1). Obel grade (20). The bold font indicated to emphasize *p* values < 0.05.

## 4. Discussion

### 4.1. Experimental model for laminitis induction and effect of treatment using buffer solution

Despite the small number of animals per group, we showed that starch overload was effective at inducing laminitis in 50% (5/10) of the animals, regardless of treatment. The partial ineffectiveness of single carbohydrate overload to induce laminitis in the other 50% of the horses can be attributed to individual differences between animals.

Regarding the ineffectiveness of the buffer solution in promoting improvement of the clinical condition in the SBG, some theories can be raised. The buffer solution was composed only of 2 weak bases (aluminum and magnesium hydroxide), without the addition of their respective salts, which may have limited its buffering potential. In a study carried out with healthy horses, the oral supplementation of a solution containing 0.2 g/kg of magnesium hydroxide and aluminum was able to significantly increase the fecal pH, up to 24 h after administration (30). In the present work, the composition of the solution was fixed (3.5 g of aluminum hydroxide and 65.6 g of magnesium hydroxide), regardless of the animal's weight. In this situation, many horses received a dose lower than 0.2 g/kg. In addition, Garner et al. (31) demonstrated that cecal pH usually drops from 7.18 to 5.72 8 h after CHO overload, and to 4.14 after 24 h. Perhaps, SBG would have benefited from a second administration of the buffer solution between T8h and T24h.

### 4.2. APP kinetics and correlation with clinical parameters during the inflammatory process linked to laminitis syndrome

α-1-antitrypsin is a major endogenous protease inhibitor, with anti-inflammatory properties that tries to contain tissue damage during acute injury (32). Serum concentration of α-1-AT was significant higher in SBG at T24h and T48h, when comparing to CG. Its 15% increase on SBG at T24h could have contributed to positive modulation of the inflammatory response, event not observed in SG (Figure 2). However, as shown in Table 4, there was a negative correlation between increased number of steps and serum levels of this protein on SG. Bearing in mind that horses that developed clinical laminitis had an increased number of steps, it can be speculated that α-1-AT behaved, in horses within SG, as an anti-inflammatory agent.

Similar elevation patterns of Trf were found by Barros et al. (33) in donkeys submitted to laparoscopic ovariectomy and by Nogueira et al. (26) in the peritoneal fluid of horses with intestinal obstruction. When evaluating the effect of treatment not associated with the inflammatory condition (BG × CG), it is noted an increase in Trf concentration in the animals treated with buffer solution, up to 16 h after its administration (T12h and T24h). Being a negative APP in most mammal species, it was expected decreasing values over time in SG and SBG, due to systemic inflammatory conditions. However, this decrease is associated to Fe^2+^ reduced absorption to reduce bacterial growth (34). Only one animal from SBG presented suggestive signs of sepsis, 48 h after administration of CHO overload, which reinforces the absence of Trf reduction in SG and SBG.

Correlations among APP, hematological, inflammatory, and systemic parameters were stablished for some diseases in equine species (35–37), including positive correlation between Obel grade and α-1-globulin in clinical laminitis (38). Despite lack of more information on searched data, we were able to demonstrate some positive correlations between APP values and clinical signs in animals subjected to starch overload in this laminitis induction model.

It is known that Cp plays an important role in the metabolism and transport of copper, and its elevation is related to the increase of reactive oxygen species, produced during inflammation (39). Oxidative stress has been directly related with the pathogenesis of laminar lesions, especially during sepsis, or when black walnut extract and hyperinsulinemia models are used to induce laminitis (40–42). In this way, this study showed that aCp was a good bioindicator during the early stages of laminitis, since its positive correlation with number of steps in SG and SBG, and Obel grade and HR in SBG.

The response pattern of serum amyloid A (SAA) in horses has been described in few diseases (13–16, 43–46). SAA concentrations varies considerably according to studies, which may suggest individual differences in the immune response and in the methods used for its quantification. Therefore, it is recommended individual interpretation, comparing SAA baseline values with those after inflammatory stimulus, injury, and treatment (45). The values represented in Table 3 demonstrate high variability (high standard deviation) in SAA concentration, which perhaps justifies the absence of a significant difference over time and between sick groups (treated and untreated). When evaluating percentage of variation (Figure 2), assuming samples collected at T0h as baseline, it is noted significant increased concentration of this protein until T72h in the groups treated with buffer solution (106% in the SBG and 62% in BG). SG showed, on the other hand, an increase of only 14%, demonstrating that the buffer solution had a greater impact in SAA expression than CHO overload. It was demonstrated elevated concentrations of amyloid A isoform 3 in synovial fluid of horses submitted to septic arthritis (47–49). These proteinases are known to be involved in physiological and morbid degradation of the extracellular matrix, being related to development of lamellar lesions in horses with laminitis (50, 51). Therefore, detection and classification of local SAA in injured sites may, in the future, increase the diagnostic potential of this protein, allowing distinction between local and systemic conditions.

The absence of a significant difference in serum CRP values corroborate previously studies (52, 53). In humans, high concentrations of this protein were found in the presence of metabolic syndrome (54, 55) and insulin resistance (56). Although ingestion of high amounts of CHO can contribute with the development of these two syndromes in horses (57), the model used to induce laminitis, in this study, consisted in a single CHO overload administration, which may justify the absence of a uniform response to the development of the syndrome, with little repercussion on the levels of CRP.

As previously described (58), horses with induced laminitis by CHO overload showed no significant changes on total serum protein values at the prodromal stage of this syndrome, corroborating our findings. Alb values, obtained through spectrophotometry, also did not vary over the experimental times (Table 3), nor between groups (*p* > 0.05). As albumin concentrations tend to decrease from the sixth day after inflammatory stimulus (59), these values were expected, since the last blood sample were collected 72 h after administration of the starch overload, and, also, only 40% (4/10) of horses developed severe systemic inflammatory condition.

## 5. Conclusions

The single administration of starch overload was able to induce clinical laminitis in 60% of SBG and 40% of SG animals. Also, the single dose of buffer solution was not effective in preventing the development of laminitis, and a booster dose should be considered in further studies. Transferrin, considered a negative APP, showed a positive response pattern in SG and SBG. Serum ceruloplasmin concentrations were positively correlated with heart rate, Obel grade and number of steps in animals from SBG. Ceruloplasmin, α-1-antitrypsin and haptoglobin concentrations increased considerably in SBG, suggesting greater activation of inflammatory response in animals of this group, probably due to the better induction of laminitis. Changes in clinical parameters were also more evident in the SBG, corroborating the protein fractionation findings.

## Data availability statement

The original contributions presented in the study are included in the article/supplementary material, further inquiries can be directed to the corresponding author.

## Ethics statement

The animal study was reviewed and approved by Ethics Committee on the Use of Animals (CEUA) of the School of Agricultural and Veterinarian Sciences—FCAV/Unesp-Jaboticabal Campus, under the protocol # 23.391/15.

## Author contributions

IP, PC, and CA: conception and design of the work. IP, VB, and CC: clinical trial execution and data collection. IP, AS, and DG: data analysis. IP: manuscript elaboration. AS, DG, and CA: manuscript review. AB: LC-MS analysis. All authors contributed to the article and approved the submitted version.
